# Some Aspects of Intestinal Obstruction

**Published:** 1908-09-19

**Authors:** 


### Surgery.

### SOME ASPECTS OF INTESTINAL OBSTRUCTION.

### THE TREATMENT OF OBSTRUCTION DUE TO CAUSES OUTSIDE THE BOWEL.

The obvious treatment is to remove the obstructing cause, if possible. If this is a simple ovarian or uterine growth the operation will be easy; but if the growth is a malignant one, which not only presses on, but actually involves the bowel, removal may be out of the question, and the establishment of an artificial anus is the only alternative left.

The treatment of obstruction from without by adhesions is complicated by the fact that surgical interference is often unsatisfactory, although when the adhesions are limited, brilliant results may be obtained. It is not always easy to separate the adhesions, and even if this is done, fresh ones are almost always quickly formed. This, then, is a class of case in which operative treatment should not be resorted to in a hurry, unless the symptoms are becoming urgent. Palliative treatment, too, sometimes confers real benefit in these cases. Careful dieting, combined with the administration of mild laxatives and abdominal massage, may in time stretch and loosen the adhesions. If the symptoms are severe, a laparotomy must, of course, be undertaken. The abdomen should be opened just to one or other side of the middle line through the rectus muscle, unless there is a very distinct indication to the contrary, as afforded by a definite history of past localised peritonitis. In this case a suitable incision may be made over the region of the original disease. Great care must be taken in opening the abdomen, because the intestines are often densely adherent to the parietal peritoneum, and may thus easily be damaged. The subsequent procedure depends entirely upon the conditions disclosed. Isolated adhesions limited to one portion of the intestine may be carefully divided, and this will often suffice to effect a cure.

When the condition is due to the traction of adhesions on a loop of bowel producing a kink, the adhesions should be divided. In some cases the lumen of the bowel is at once restored, but more frequently there are also found adhesions between the two limbs of the kink which prevent the bowel from returning to its normal state. If it is impossible to break these down, the kinked loop must be excised and the bowel united by end-to-end anastomosis.

When the bowel is adherent to the parietal peritoneum, it is better to cut the adherent portion free from the rest of the peritoneum and leave it attached to the bowel, rather than to try to separate it, since any attempt to do this may tear the gut if the adhesions are firm, as they nearly always are.

Cases in which a large mass of intestine is matted together are always difficult. It is not a good practice to attempt to unravel the coils, even in those rare cases where it appears possible, since new adhesions will quickly form and produce a recurrence of the obstruction. The choice here lies between a lateral anastomosis, and an excision with an end-to-end anastomosis. Either procedure is of course a very serious undertaking, but the latter operation is undoubtedly the more severe of the two. At the same time it is a better procedure than lateral anastomosis, which leaves in the abdomen a mass of intestine in which bowel contents will accumulate and undergo decomposition. The choice, therefore, should be decided by the condition of the patient. If his condition will stand it, resection should be undertaken, but if not, the surgeon must be content with doing a lateral anastomosis. If there is any doubt as to the extent to which surgical interference is justifiable it is safest to be guided by the rule that the least possible amount of manipulation is advisable, provided that temporary relief of the obstruction is obtained. Any attempt to deal with massive adhesions in the direction of breaking them down is likely to lead to their re-formation, and the occurrence of a similar, and possibly more serious, attack of obstruction in the future. Whereas, if the actual obstruction is overcome at the moment, it will be found that the peritoneum will in the course of time deal with the adhesions more successfully than any surgeon can ever hope to do. The truth of this will be borne in on anyone who has had occasion at a subsequent operation to inspect the interior of a peritoneal cavity which had been the seat of adhesions. In some cases the altered state of affairs is almost past belief, and if one had not known the previous history of the case, one would hardly be able to credit the fact that there had ever been any adhesions at all. This is especially remarkable in cases in which the original trouble had been due to tuberculous peritonitis of the adhesive variety. The writer remembers one such case. The patient was a young girl who had symptoms of chronic intestinal obstruction, and a mass in the right iliac fossa. The abdomen was opened and it was found that the cæcum and the last few inches of the small intestine were involved in a dense mass of adhesions, due to tuberculous peritonitis. A lateral anastomosis was made between the ileum and ascending colon, a Murphy's button being used. The immediate result was satisfactory, but the button never passed, and could be seen by means of $x$-rays in its original position. A second operation was performed some months later, with the object of dislodging the button, and there was then no intra-peritoneal evidence of any past inflammatory condition at all.

The treatment of obstruction due to causes in the lumen of the bowel, such as an enterolith or foreign body, is comparatively simple. The foreign body must be removed by operation. Two precautions should be taken: the gut should be opened at its anti-mesenteric border, and not immediately over the foreign body, but either just above or just below. The reason of this is that the bowel is usually inflamed at the point where the body is impacted, and the sutures may cut out. A single row of Lembert sutures is used to close the intestine.

